# Honey Bee Suppresses the Parasitic Mite Vitellogenin by Antimicrobial Peptide

**DOI:** 10.3389/fmicb.2020.01037

**Published:** 2020-05-25

**Authors:** Yunfei Wu, Qiushi Liu, Benjamin Weiss, Martin Kaltenpoth, Tatsuhiko Kadowaki

**Affiliations:** ^1^Department of Biological Sciences, Xi’an Jiaotong-Liverpool University, Suzhou, China; ^2^Department for Evolutionary Ecology, Institute for Organismic and Molecular Evolution, Johannes Gutenberg University Mainz, Mainz, Germany

**Keywords:** host-parasite/pathogen interaction, vector-pathogen interaction, honey bee, parasitic mite, deformed wing virus, antimicrobial peptide, Vitellogenin

## Abstract

The negative effects of honey bee parasitic mites and deformed wing virus (DWV) on honey bee and colony health have been well characterized. However, the relationship between DWV and mites, particularly viral replication inside the mites, remains unclear. Furthermore, the physiological outcomes of honey bee immune responses stimulated by DWV and the mite to the host (honey bee) and perhaps the pathogen/parasite (DWV/mite) are not yet understood. To answer these questions, we studied the tripartite interactions between the honey bee, *Tropilaelaps mercedesae*, and DWV as the model. *T. mercedesae* functioned as a vector for DWV without supporting active viral replication. Thus, DWV negligibly affected mite fitness. Mite infestation induced mRNA expression of antimicrobial peptides (AMPs), Defensin-1 and Hymenoptaecin, which correlated with DWV copy number in honey bee pupae and mite feeding, respectively. Feeding *T. mercedesae* with fruit fly S2 cells heterologously expressing honey bee Hymenoptaecin significantly downregulated mite *Vitellogenin* expression, indicating that the honey bee AMP manipulates mite reproduction upon feeding on bee. Our results provide insights into the mechanism of DWV transmission by the honey bee parasitic mite to the host, and the novel role of AMP in defending against mite infestation.

## Introduction

Large-scale loss of managed honey bee colonies has been recently reported across the globe (Goulson et al., 2015). Since pollination by honey bees is vital for maintaining ecosystems and the production of many crops (Klein et al., 2007; Aizen and Harder, 2009), prevention of honey bee colony losses has become a major focus in both apiculture and agriculture. Colony losses have often been associated with the ectoparasitic mites *Varroa destructor* and *Tropilaelaps mercedesae*, which feed on honey bees and transmit honey bee viruses, particularly deformed wing virus (DWV) to the host (de Miranda and Genersch, 2010; Rosenkranz et al., 2010; Chantawannakul et al., 2018). In the absence of mites, DWV copy numbers remain low in honey bees without specific symptoms (covert infection). However, DWV levels associated with honey bees are dramatically increased in mite infested colonies (Shen et al., 2005; Forsgren et al., 2009; Khongphinitbunjong et al., 2015; Wu et al., 2017). These honey bees often show multiple symptoms (overt infection), which include the death of pupae, deformed wings, shortened abdomen, and reduced lifespan (Yue and Genersch, 2005; Tentcheva et al., 2006; de Miranda and Genersch, 2010; Rosenkranz et al., 2010). Winter colony loss is strongly correlated with the presence of DWV and *V. destructor* (Highfield et al., 2009; Nazzi and Le Conte, 2016).

Although the impacts of DWV and *V. destructor* on individual honey bees and colonies are well characterized, the actual relationship between DWV and honey bee mite is not yet understood. Several studies have suggested that DWV replicates in *V. destructor* and that more virulent DWV strains are amplified for transmission to honey bees (Martin et al., 2012; Ryabov et al., 2014). However, the results of other studies are inconsistent with this view (Erban et al., 2015; Dong et al., 2017; Posada-Florez et al., 2019). Thus, it is important to address this issue to uncover the mechanism by which mites function as vectors for DWV.

DWV copy numbers in mites can exceed 10^6^ (Wu et al., 2017; Posada-Florez et al., 2019). Thus, DWV could have significant effects on mite physiology. Previous studies have reported that DWV infection and/or *V. destructor* infestation induce honey bee immune responses that include synthesis of antimicrobial peptides (AMPs) (Gregorc et al., 2012; Kuster et al., 2014). However, their effects on the host (honey bee), pathogen (DWV), and parasite (mite) are still uncharacterized. AMPs were originally identified as short positively-charged peptides that inhibit the viability of bacteria and fungi (Bahar and Ren, 2013; Hanson and Lemaitre, 2019). Since AMPs are induced under various conditions, their physiological functions could be more diverse and remain to be tested.

In this study, we first examined whether *T. mercedesae* functions as a bona fide vector for DWV, and then characterized the effects of DWV on mites to understand the precise relationship. We also examined the immune responses of honey bee pupae to *T. mercedesae* infestation and found that Hymenoptaecin down-regulates the mite *Vitellogenin* (*Vg*) gene. Using these results, we discuss the mechanisms of DWV transmission by the honey bee parasitic mites, as well as how the host (honey bee) defends against the parasite (mite) by suppressing reproduction.

## Materials and Methods

### Artificial Infestation of Honey Bee Pupae With Mites

*A. mellifera* colonies were obtained from local beekeepers and maintained at Xi’an Jiaotong-Liverpool University. Honey bee worker pupae (*n* = 33) with white eyes were sampled from the mite-free colony by opening the capped brood cells. Adult female mites (*n* = 15) were collected from another colony heavily infested with *T. mercedesae* as above. The average copy number of DWV in the mite infested pupae was 6.2 × 10^7^. A single pupa and mite were put inside a gelatin capsule. As the control, the remaining pupae (*n* = 18) were individually incubated without the mite. The capsules were inserted to a tube rack vertically positioned in an incubator at 33°C with 70% relative humidity for a week (Egekwu et al., 2018).

### Isolation of Total RNA and RT-PCR

Head was first dissected from each pupa and total RNA was extracted from the individual pupal heads and mites using TRI Reagent^®^ (Sigma-Aldrich) according to the manufacturer’s instruction. Glycogen (1 μg) was added to facilitate isopropanol precipitation of the mite RNA sample. Reverse transcription (RT) reaction was carried out using 1 μL of total RNA, random primer (TOYOBO), ReverTra Ace (TOYOBO), and RNase inhibitor (Beyotime). RNase H (Beyotime) was then added to digest RNA in RNA/cDNA heteroduplex after cDNA synthesis. DWV in the honey bee and mite samples was detected by RT-PCR using DWV #1 primers (Supplementary Table S1) and the cycling condition of 2 min at 94°C followed by 32 cycles of 10 sec at 98°C, 20 sec at 55°C, and 30 sec at 68°C. The PCR products were analyzed by 2% agarose gel. *A. mellifera* and *T. mercedesae EF-1*α mRNAs (Supplementary Table S1) were used as the positive controls to verify successful RT.

### Sequencing of RT-PCR Products

PCR products obtained by above RT-PCR were purified by QIAquick PCR Purification Kit (QIAGEN) according to the manufacturer’s instruction, and then directly sequenced by Sanger method using the PCR primers.

### Analysis of DWV, Honey Bee *AMP*, and Mite *Vg* mRNAs by qRT-PCR

DWV copy number was determined by qRT-PCR using a Hieff^TM ®^ qRT-PCR SYBR Green Master Mix (Low Rox Plus, Yesen) and DWV #2 primers (Supplementary Table S1). To prepare a standard curve for DWV, PCR product obtained by above primers was purified and the copy number was determined by a formula below.

$$\mathrm{Copy}\,\mathrm{number}=\,\frac{\begin{array}{c} \mathrm{DNA}\,\mathrm{concentration}\,\left(\text{ng}/\mu \,l\right)\,\times \,6.02\,\\ \times 10^{23}\,\,\left(\text{copies}/\text{mol}\right) \end{array}}{\text{Length}\,\left(\text{bp}\right)\,\times \,6.6\,\times 10^{11}\,\,\left(\text{ng}/\text{mol}\right)}$$

6.6 × 10^11^ ng/mol is the average molecular mass of one base pair and 6.02 × 10^23^ copies/mol is Avogadro’s number. We conducted qPCR using 10^1^–10^9^ copy number of the PCR product and then plotted the Ct values against the log values of copy numbers. DWV copy number in the sample was determined using the standard curve. The amount of cDNA added to each qPCR reaction was normalized using either *A. mellifera* or *T. mercedesae 18S rRNA* as the endogenous reference (Supplementary Table S1).

The relative amounts of *Hymenoptaecin*, *Defensin-1*, and *T. mercedesae Vg* mRNAs in the samples were measured by the ΔΔCt method. The primers for *Hymenoptaecin*, *Defensin-1*, and five *T. mercedesae Vgs* are listed in Supplementary Table S1. *A. mellifera* or *T. mercedesae 18S rRNA* was used as the endogenous reference.

### Effects of Introducing Wound on DWV Copy Numbers in Honey Bee Pupae

The mite-free honey bee pupae with pink eyes were sampled from *T. mercedesae-*infested colony. A small physical wound was introduced to the thorax (*n* = 6) with a sterilized microliter syringe (GAOGE) and control pupae (*n* = 7) were untreated. All pupae were then individually put in a gelatin capsule and incubated for 37 h and DWV copy numbers in the individual pupal heads were measured as above.

### Raising Antibodies Against VP1 and RdRP of DWV

The P-domain of VP1 (amino acid 748-901 of DWV polyprotein) was PCR amplified using 5′-*Nde*I-P-domain and 3′-*Xho*I-P-domain primers (Supplementary Table S1). The PCR amplicon was digested with *Nde*I (NEB) and *Xho*I (NEB). The part of RdRP (amino acid 2563–2797 of DWV polyprotein) was also PCR amplified using 5′-*Kpn*I-RdRP and 3′-Hind III-RdRP primers (Supplementary Table S1). The PCR product was digested with *Kpn*I (NEB) and Hind III (NEB). The restriction enzyme digested DNA fragments were purified and then cloned to the corresponding sites in pCold-I vector (TAKARA) followed by transformation to *E. coli* BL21.

The transformed *E. coli* was grown in LB medium containing ampillicin (0.1 mg/mL) at 37°C until the A_600_ reached to approximately 0.5, and then the culture was cooled down and added with isopropyl-thio-galactoside at the final concentration of 0.5 mM to induce the protein expression at 15°C overnight. *E. coli* was suspended with 100 mL of lysis buffer (50 mM NaH_2_PO_4_, 300 mM NaCl, 1 mM DTT, and protease inhibitors at pH 8.0). The cell suspension was then sonicated for 45 min (30 sec sonication with 3 min interval) at amplitude 100 on ice using Q700 sonicator (Qsonica). After centrifugation, the supernatant was collected and incubated with His-tag Protein Purification Resin (Beyotime) at 4°C for 2 h. The resin was then washed five times with 10 mL of washing buffer (50 mM NaH_2_PO_4_, 300 mM NaCl, and 2 mM imidazole, pH 8.0), and the bound protein was eluted six times with 1 mL then twice with 5 mL of elution buffer (50 mM NaH_2_PO_4_, 500 mM NaCl, and 250 mM imidazole, pH 8.0). The purified proteins were dialyzed against PBS at 4°C overnight, and then concentrated using Vivaspin^®^ 6 polyethersulfone 10 kDa (Sartorius). Concentrations of the purified proteins were measured using BCA protein assay kit (Beyotime) according to the manufacturer’s instruction. Purification of the RdRP peptide was carried out using above buffers containing 0.1% sarcosyl to increase the protein solubility. The purified proteins were delivered to a company (GeneScript-Nanjing) to obtain the affinity purified rabbit polyclonal antibodies.

### SDS-PAGE and Western Blot

The proteins were suspended with the sample buffer (2% SDS, 10% glycerol, 10% β-mercaptoethanol, 0.25% bromophenol blue, and 50 mM Tris-HCl, pH 6.8), and then heated at 99°C for 5 min followed by applying to 12% SDS-PAGE gel. After electrophoresis, the gel was treated with the staining buffer (0.25% Coomassie brilliant blue G-250, 40% methanol, and 10% acetic acid), and then washed with the destaining buffer (40% methanol and 10% acetic acid). The bands were visualized and analyzed using ChemiDoc^TM^ MP imaging system and Image Lab^TM^ touch software (BIO-RAD).

Honey bee pupal heads and *Tropilaelaps* mites were individually homogenized with 300 and 50 μL of sample buffer, respectively. *Drosophila* S2 cells in 12-well plate were lysed with 200 μL of sample buffer. All samples were heated as above and centrifuged, and then the supernatants were applied to 10 (for honey bee and mite lysates) or 15% (for S2 cell lysates) SDS-PAGE gel. After electrophoresis, proteins in the gel were transferred to a pure nitrocellulose blotting membrane (Pall^®^ Life Sciences). The membrane with proteins was first blocked with PBST (0.1% Tween-20/PBS) containing 5% BSA at room temperature for 1 h, and then incubated with 1000-fold diluted primary antibody (anti-VP1 antibody, anti-RdRP antibody, or anti-His tag antibody) in above buffer at 4°C overnight. The membrane was washed with PBST three times for 5 min each, and then incubated with 10,000-fold diluted IRDye^®^ 680RD donkey anti-rabbit IgG (H + L) (LI-COR Biosciences) in PBST containing 5% skim milk at room temperature for 1.5 h. The membrane was washed as above, and then visualized using Odyssey Imaging System (LI-COR Biosciences).

### Analysis of Mite Transcriptomes by RNA-Seq

Individual *Tropilaelaps* mites were tested for DWV by RT-PCR as above and separated to two groups with either high (High_A and High_B) or low (Low_A and Low_B) DWV level. Each group was made of total RNAs prepared from ten mites and sequenced at BGI (Shenzhen, China) using Illumina HiSeq 4000 platform. After sequencing, the raw data were filtered to remove the adaptor sequences, contamination, and low-quality reads by BGI. The Quality control (QC) was further analyzed using FastQC. All RNA-seq data are available in SRA database with the accession #: PRJNA608093.

The reference genome and annotated genes of *T. mercedesae* were first acquired from NCBI^1^, and then used for building the index by Hisat2–build indexer (Kim et al., 2015). The generated index files were used to align the clean reads of four RNA-seq samples to the reference genome. Subsequently, SAM file outputs from the previous step were sorted using SAMtools (Li et al., 2009). HTSeq-count (Anders et al., 2015) was further applied to obtain the raw read counts for downstream analysis of identifying the differentially expressed genes (DEGs) between the mites with low and high DWV loads in *R* (V3.4.3) based Bioconductor edgeR package (V3.20.9) (Robinson et al., 2010). The DEGs were cut-off by a False Discovery Rate (FDR) at 0.05.

### Immunostaining of the Mite Thin Sections

*Tropilaelaps* mites collected from the hive were washed with bleach and PBS, and then fixed with 4% paraformaldehyde/PBS at 4°C for 24 h with gentle shaking. The fixed samples were stored in methanol at −20°C and then converted into absolute n-butanol followed by embedding in Technovit 8100 (Kulzer, Wehrheim, Germany). On the rotation microtome (Leica RM 2245), 8 μm thick serial sections were prepared with glass knives and placed into water drops on silanized slides. The sections were dried for 30 min at 50°C. The above thin sections were washed five times with PBS, and then treated with 2 mg/mL pepsin in 0.9% NaCl, pH 2.0 at 37°C for 10 min. They were then washed three times with PBS, three times with PT (PBS containing 0.1% Triton-X 100), and once with PBS. The sections were blocked with PBS containing 3% BSA and 1% normal goat serum at 4°C overnight and then they were alternatively incubated with 1,000-fold diluted anti-VP1 antibody and the pre-immune serum in above buffer at 4°C overnight. After washing eight times with PBS, the sections were incubated with 1,000-fold diluted Goat anti-rabbit IgG (H + L) Superclonal^TM^ secondary antibody, Alexa Fluor 555 (Thermo Scientific) at room temperature for 1.5 h. After washing seven times with PBS, the sections were incubated with 0.5 μg/mL DAPI (Beyotime) at room temperature for 15 min. Following the final wash with PBS, the sections were mounted with Antifade mounting medium (Beyotime). Each wash was conducted for 10 min. The immunostained sections above were observed using a confocal microscope, LSM 880 (Zeiss) with the TileScan method. ImageJ was used for the image analysis.

### Phylogenetic Trees of the DWV Isolates and Mite Vgs

The representative RNA sequences of DWV isolates from the honey bee pupae and mites were aligned using MUSCLE (Edgar, 2004), and then Tamura 3-parameter + G was selected as the best-fit substitution model for constructing the phylogeny. The condensed phylogenetic tree was constructed using the maximum likelihood method and a bootstrap value of 1,000 replicates with MEGA7 (Kumar et al., 2016). Amino acid sequences of all *T. mercedesae* and *V. destructor* Vgs were retrieved from NCBI and the phylogenetic tree was constructed as above except Jones-Taylor-Thornton + G + F was used as the best model.

### Codon Usage Analysis

Codon usage tables to show the codon frequencies per 1000 codons for DWV, *A. mellifera*, and *T. mercedesae* were obtained using HIVE-CUTs (Athey et al., 2017). To compare the codon frequencies between DWV and either *A. mellifera* or *T. mercedesae*, three stop codons, and codons for methionine and tryptophan were omitted from the analysis.

### Establishing *Drosophila* S2 Cells to Stably Express Hymenoptaecin

*Hymenoptaecin* cDNA was obtained by RT-PCR using honey bee RT and Hymenoptaecin #1 primers (Supplementary Table S1). The PCR amplicon was digested with *Eco*RI and *Age*I and cloned to the same sites in pAc5.1/V5-His B (Thermo Fisher Scientific) to express the His-tagged protein. S2 cells in 12-well plate were transfected with either 2 μg of Hymenoptaecin expression vector or empty vector and 10 μL of HilyMax (DOJINDO) for 24 h. After replacing the medium, the cells were cultured for 24 h, and then analyzed by western blot using rabbit polyclonal anti-His tag antibody (BBI). After confirming the protein expression, the untagged version of *Hymenoptaecin* cDNA was PCR amplified using Hymenoptaecin #2 primers (Supplementary Table S1) and digested with *Xho*I and *Eco*RV followed by inserting to the same sites in pMK33/pMtHy (Koelle et al., 1991). S2 cells were transfected with this expression vector or empty pMK33/pMtHy as above and the stable transfectants were first selected by 0.1 mg/mL hygromycin for 10 days, and then the concentration was increased to 0.3 mg/mL. The hygromycin-resistant S2 cells (2 × 10^7^) were harvested during the logarithmic growth phase and suspended with 0.3 mL of Grace medium followed by sonication and store at −20°C.

### Feeding Mites With S2 Cell Extracts

Feeding *Tropilaelaps* mites was carried out in an inverted flat-cap 2 mL microcentrifuge tube. The bottom of the tube was cut and plugged with cotton to provide ventilation and the cap contained a small piece of a sterile cotton ball. S2 cell extracts prepared as above together with 2% Royal blue (50 μL) were dispensed onto the cotton and then 10 starved mites were added prior to closing the tube. The assay tubes were placed in an incubator at 33°C with 90% relative humidity for 24 h under dark. The fed and survived mites were collected and then five *Vg* mRNAs were analyzed by qRT-PCR.

### Statistical Analysis

Statistical analyses were performed with BellCurve for Excel (Social Survey Research Information Co., Ltd.) and no data point was excluded. To compare the statistical difference between two groups, we used Brunner–Munzel test, a non-parametric method applicable to samples with unequal variance. To test correlation between two factors, Pearson correlation test was used. All data presented were from representative independent experiments. The applied statistical tests and *P*-values are also described in figure legends.

## Results

### Role of *T. mercedesae* as a Vector for DWV

**FIGURE 1:**
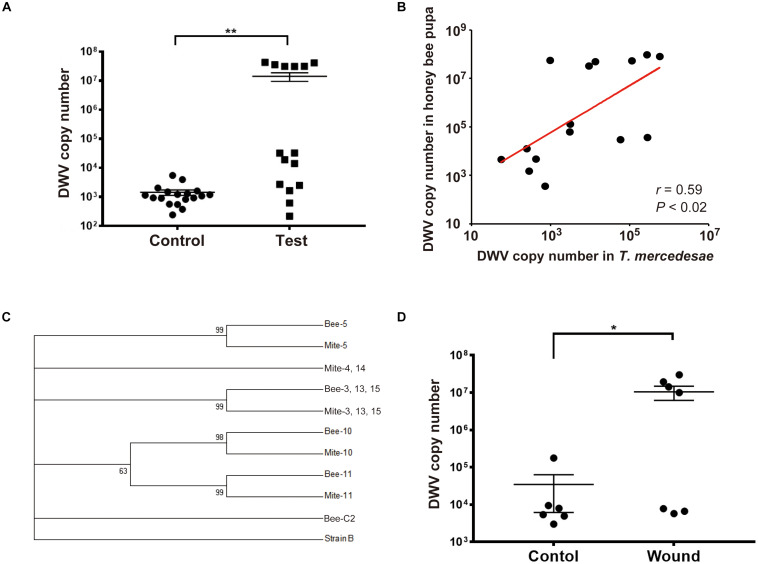
*T. mercedesae* functions as a vector for DWV. **(A)** DWV copy numbers in the heads of honey bee pupae artificially infested by *T. mercedesae* (Test, *n* = 15) and the ones without mites (Control, *n* = 18). Mean values with error bars (± SEM) are indicated. Two groups are statistically different by Brunner–Munzel test (**P < 1.3E-06). **(B)** DWV copy numbers in individual honey bee pupae and the infesting *T. mercedesae* are plotted on the Y- and X-axis, respectively. The red line represents a fitted curve and the Pearson correlation value and *P*-value are also shown. **(C)** Maximum-likelihood condensed phylogeny of DWV isolates from honey bee pupae (Bee) and *T. mercedesae* (Mite) was constructed based on partial VP2 and VP1, and full VP4 sequences. Sequence of Bee-C2 (control pupa) sample is identical to those of other seven control pupae (Bee-C6, 9, 10, 11, 14, 15, and 16), seven test pupae (Bee-1, 4, 7, 8, 9, 12, and 14) and five infesting mites (Mite-1, 7, 8, 9, and 12). Bootstrap values are shown at the corresponding node of each branch and DWV type B strain was used as an outgroup. **(D)** DWV copy numbers in wounded (Wound, *n* = 7) and untreated (Control, *n* = 6) honey bee pupae. Mean values with error bars (± SEM) are indicated. Two groups are statistically different by Brunner–Munzel test (*P < 0.041).

To directly test whether *T. mercedesae* functions as a vector to transmit DWV to honey bees, we individually incubated white-eyes worker pupae with *T. mercedesae*. The heads of test pupae (the ones artificially infested by the mites) contained higher DWV copy numbers than those of the control pupae (Figure 1A). We also found a positive correlation between DWV copy number in pupae and infesting mites (Figure 1B). Sanger sequencing of the PCR amplicons (Supplementary Figure S1) revealed that ten pupa-mite pairs were infected by a single variant of DWV. Two pairs were infected by multiple variants in both pupae and mites. Two pairs were infected by single and multiple variants in pupae and mites, respectively. One pair was infected by single and multiple variants in mite and pupa, respectively (Supplementary Table S2). A phylogenetic tree was constructed using DWV sequences obtained from test pupae, infesting mites, as well as the control pupae. In case multiple DWV variants are present in pupae and mites, only samples in which we could identify the dominant variants were analyzed. All 18 control pupae were infected by multiple variants at very low level and the eight samples (Bee-C2, 6, 9, 10, 11, 14, 15, and 16) contained the dominant DWV variant, which was identical to the one present in seven test pupae (Bee-1, 4, 7, 8, 9, 12, and 14) and five infesting mites (Mite-1, 7, 8, 9, and 12). Three pupa-mite pairs (Bee/Mite-3, 13, and 15) contained the same variant. Mite-4 and Mite-14 shared the same variant, which was different from the one present in the infested pupae (Bee-4 and Bee-14). These two pairs represent the example that pupa and the infesting mite do not share the same DWV variant. As shown in Figure 1C, six pupa-mite pairs (Bee/Mite-3, 5, 10, 11, 13, and 15) out of 15 pairs were infected by four variants that were different from the ones present in the control pupae (Fisher’s exact test*; P* < 0.005). These results demonstrated that these variants were transferred from the infesting mites derived from a colony that was different from the one in which all pupae were sampled. To test whether *T. mercedesae* also enhances replication of the endogenous DWV by inflicting injury, we induced wounds at the pupal thorax with a sterile needle and compared the copy numbers of DWV in the heads of wounded and control (untreated) pupae. The copy numbers were higher in the wounded pupae than in the control pupae (Figure 1D), suggesting that wound induction alone is sufficient to stimulate replication of the endogenous DWV in honey bee pupae.

### Impact of DWV on *T. mercedesae*

**FIGURE 2:**
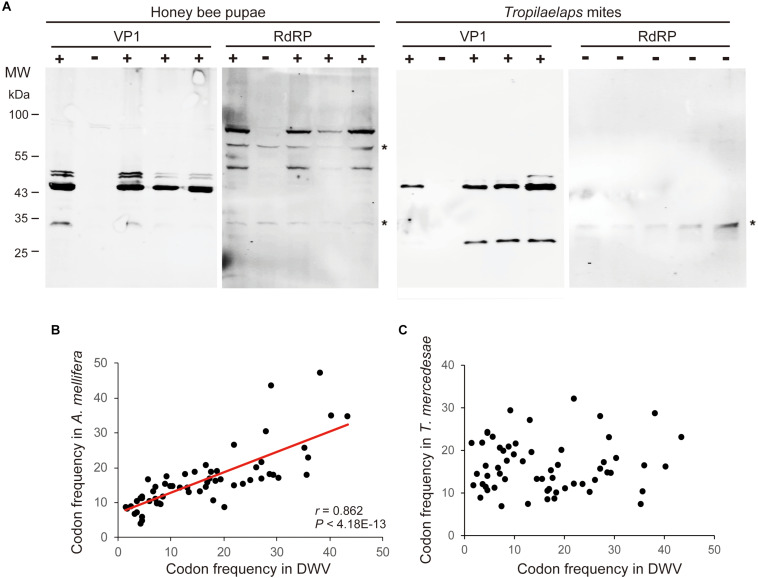
Lack of active replication of DWV in *T. mercedesae.* **(A)** Detection of DWV VP1 and RdRP in the lysates of individual honey bee pupae and *Tropilaelaps* mites by western blot. A major 47 kDa band was detected in VP1-posiitive (+) but not negative (−) pupa and mite; however, RdRP (90 and 53 kDa bands) was only present in the VP1-positive pupa. The asterisks represent non-specific proteins cross reacted with anti-RdRP antibody. The size of protein molecular weight marker (MW) is at the left. **(B)** Frequencies of each codon out of 1,000 codons in DWV and *A. mellifera* genomes are plotted on the X- and Y-axis, respectively. The red line represents a fitted curve and the Pearson correlation value and *P*-value are also shown. **(C)** Frequencies of each codon out of 1,000 codons in DWV and *T. mercedesae* genomes are plotted on the X- and Y-axis, respectively.

**FIGURE 3:**
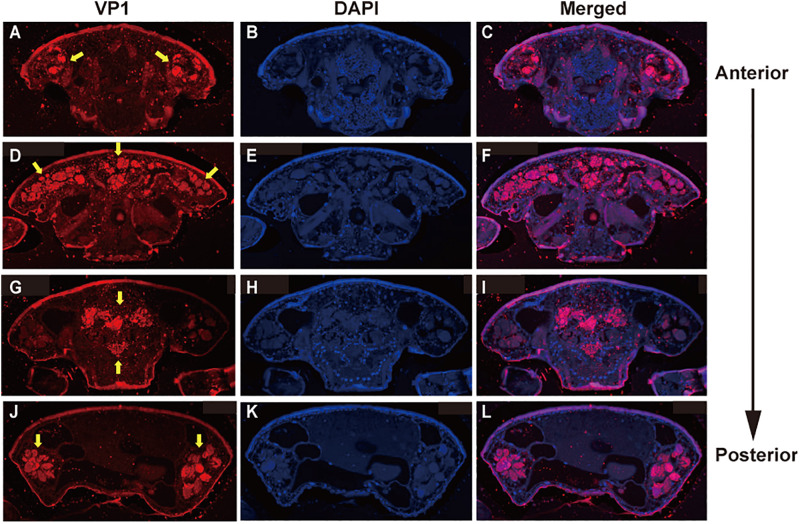
Localization of DWV in *T. mercedesae.* Transverse sections of *T. mercedesae* were immunostained by anti-VP1 antibody (VP1: **A,D,G,J**) as well as DAPI **(B,E,H,K)**. The merged images (Merged: **C,F,I,L**) are also shown. VP1-positive large dense spheres are indicated by yellow arrows. The dorsal side of mite is up and the anterior to posterior direction of mite body is also shown.

To examine the effects of DWV on *T. mercedesae*, we compared the gene expression profiles of mites carrying either high or low DWV copy numbers by RNA-seq. The average mapping rates of RNA-seq reads derived from the mites with high and low DWV loads to *T. mercedesae* genomes were 38.7 and 83.0%, respectively, and those to the DWV genome were 22.8 and 0.05%, respectively (Supplementary Table S3). We identified a few DEGs between mites with low and high DWV loads (Supplementary Table S4). The high DWV load had little effect on the transcriptome profile of *T. mercedesae*, suggesting that DWV does not actively infect/replicate the mite cells. To test this hypothesis, we analyzed the protein extracts of individual honey bee pupal heads and *Tropilaelaps* mites to detect VP1 (capsid protein, structural protein) and RdRP (RNA dependent RNA polymerase, non-structural protein) of DWV. As shown in Figure 2A, two bands of 90 and 53 kDa were specifically detected by anti-RdRP antibody, suggesting that the large and small bands represent the precursor of RdRP fused with 3C-protease (3C-Pro) and the matured RdRP, respectively. The cleavage between 3C-Pro and RdRP appears to be rate-limiting for DWV, similar to other picornaviruses (Jiang et al., 2014). RdRP and the precursor were detected in the four VP1-positive, but not one VP1-negative honey bee pupal heads. However, these proteins were absent in the four *Tropilaelaps* mites, irrespective of the presence of VP1 (Figure 2A). In total, RdRP was detected in seven VP1-positive honey bee pupae but not in eight VP1-positive mites. These results indicated that DWV infects and actively replicates in the honey bee pupal head, but not the mite cells. Lack of active synthesis of DWV protein in *Tropilaelaps* mites was also supported by their large differences in codon usage. The codon usage of DWV is apparently adapted to that of the original host, *A. mellifera*, as previously reported (Chantawannakul and Cutler, 2008), but quite different from that of mite (*r* = 0.046, *P* < 0.73) (Figures 2B,C). To uncover the major sites for localization of DWV in *Tropilaelaps* mite, transverse thin sections of mites were immunostained by anti-VP1 antibody. DWV was primarily localized in the lumen of the entire midgut as large dense spheres (Figure 3), consistent with the lack of active viral replication. There was no specific staining with the pre-immune serum (Supplementary Figure S2).

### *Hymenoptaecin* and *Defensin-1* mRNAs Are Induced in Honey Bee Pupae by Infestation of *T. mercedesae*

**FIGURE 4:**
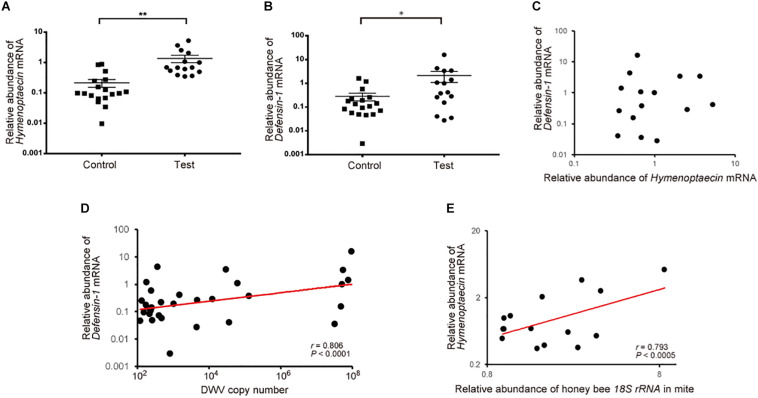
Increase of *Hymenoptaecin* and *Defensin-1* mRNAs by *T. mercedesae* infestation. Relative amounts of *Hymenoptaecin* **(A)** and *Defensin-1* **(B)** mRNAs in honey bee pupae artificially infested by *T. mercedesae* (Test, *n* = 15) and the ones without mites (Control, *n* = 18). Mean values ± SEM (error bars) are shown. Brunner–Munzel test was used for the statistical analysis (*Hymenoptaecin*: **P < 3.1E-08; *Defensin-1*: *P < 0.042). **(C)** Relative amounts of *Hymenoptaecin* and *Defensin-1* mRNAs in individual honey bee pupae are plotted on the X- and Y-axis, respectively. **(D)** DWV copy number and relative amount of *Defensin-1* mRNA in individual honey bee pupae are plotted on the X- and Y-axis, respectively. The red line represents a fitted curve and the Pearson correlation value and *P*-value are also shown. **(E)** Relative amounts of *Hymenoptaecin* mRNA in individual honey bee pupae and honey bee *18S rRNA* in the infesting mites are plotted on the Y- and X-axis, respectively. The red line represents a fitted curve and the Pearson correlation value and *P*-value are also shown.

To understand the effects of *T. mercedesae* infestation on honey bees, we quantified mRNAs of two AMPs, Hymenoptaecin and Defensin-1, in the above pupae artificially infested with *T. mercedesae* and the control (uninfested) pupae. We focused on Defensin-1 and Hymenoptaecin because they are induced in pupae by *V. destructor* infestation (Kuster et al., 2014) and under the control of the Toll and Imd pathways, respectively (Schlüns and Crozier, 2007; Lourenço et al., 2018). Thus, they represent the downstream effectors activated by Toll and Imd pathways. As shown in Figures 4A,B, both *Hymenoptaecin* and *Defensin-1* mRNAs were increased by mite infestation. However, there was no significant correlation between the amounts of *Hymenoptaecin* and *Defensin-1* mRNAs expressed in individual mite infested pupae (*r* = −0.08, *P* < 0.78; Figure 4C), suggesting that these AMPs were induced by different mechanisms during mite infestation. We then tested the correlation of DWV copy numbers and the amounts of either *Hymenoptaecin* or *Defensin-1* mRNA in both control and test pupae. The amount of *Defensin-1*, but not *Hymenoptaecin* (*r* = 0.141, *P* < 0.45) mRNA, was positively correlated with DWV copy number (Figure 4D), suggesting that *Defensin-1* is induced by DWV infection and replication. We next measured the relative amounts of honey bee *18S rRNA* in the individual mites to determine the degree of feeding of honey bee cells in fat body (Ramsey et al., 2019) and hemolymph. A positive correlation was evident between the ingestion of honey bee cells by the mite and the amount of *Hymenoptaecin* mRNA, but there was no correlation for *Defensin-1* mRNA (*r* = 0.4648, *P* < 0.83; Figure 4E).

### Hymenoptaecin Down-Regulates *T. mercedesae Vg* Gene

**FIGURE 5:**
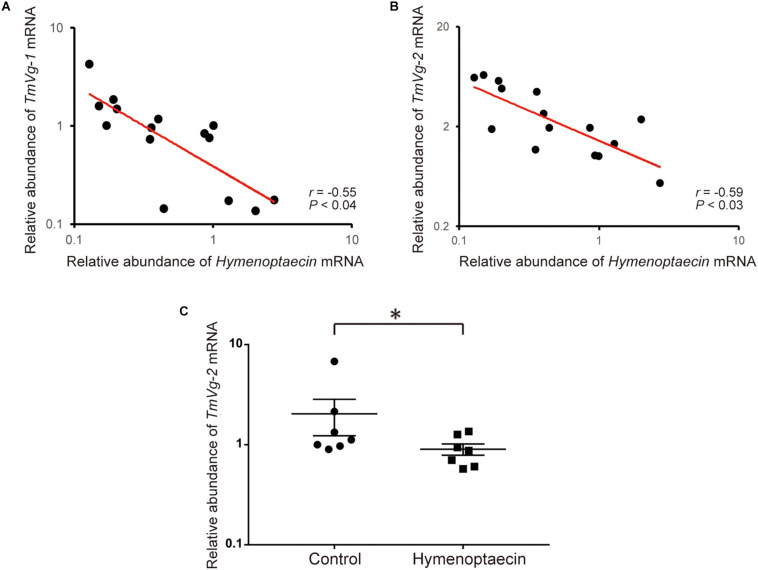
Hymenoptaecin down-regulates *T. mercedesae Vg* gene. Relative amounts of *Hymenoptaecin* mRNA in individual honey bee pupae and *TmVg-1* **(A)** or *TmVg-2* mRNA **(B)** in the infesting mites are plotted on the X- and X-axis, respectively. The red line represents a fitted curve and the Pearson correlation value and *P*-value are also shown. **(C)** Relative amounts of *TmVg-2* mRNA in individual mites fed with the extracts of S2 cells expressing *Hymenoptaecin* or wild type S2 cells (Control). Mean values ± SEM (error bars) are shown. Brunner–Munzel test was used for the statistical analysis (^∗^*P* < 0.025).

Since immune effector molecules, such as AMPs, are induced in honey bee pupae by *Tropilaelaps* mite infestation, the molecules could affect the physiology of the pupa, but also the mite, by feeding on the fat body and other tissues. *Vg* mRNA decreases with high load of DWV in *Tropilaelaps* mite by the transcriptome analysis (Supplementary Table S4). Five *Vg* related genes were identified in *T. mercedesae* genome (Accession numbers: OQR67440.1, OQR68606.1, OQR72029.1, OQR72561.1, and OQR79705.1). Based on the phylogenetic tree of *V. destructor* and *T. mercedesae* Vgs, OQR72561.1, OQR68606.1, and OQR79705.1 appeared to be *T. mercedesae* orthologs of *V. destructor* Vg-1, Vg-2, and XP_022657753.1, respectively. OQR72029.1 and OQR67440.1 were related to *V. destructor* Vg-1 and Vg-2, respectively (Supplementary Figure S3). We tested the correlation between the amounts of the five *Vg* mRNAs in *T. mercedesae* and either *Hymenoptaecin* or *Defensin-1* mRNA in mite infested pupae. There was no correlation between *Defensin-1* mRNA and any of the five *Vg* mRNAs (Supplementary Table S5). However, a negative correlation was evident between *Hymenoptaecin* mRNA and *TmVg-1* or *TmVg-2*, but not between other *Vg* mRNAs (Figures 5A,B and Supplementary Table S5). These results suggest that expression of *T. mercedesae Vg* genes is downregulated by honey bee Hymenoptaecin. To test this hypothesis, we first established *Drosophila melanogaster* S2 cells expressing honey bee *Hymenoptaecin* (Supplementary Figure S4), and then fed *T. mercedesae* with the extracts of S2 cells with or without Hymenoptaecin as previously described for *V. destructor* (Cabrera et al., 2017). The amounts of the five *Vg* mRNAs were measured in individual mites and then compared. The experiments were repeated twice and the percentages of fed/survived mites were 35 (mean) ± 7.1 (SD) % for both treatments. Consistent with the above results, *TmVg-2* mRNA was significantly reduced in mites fed with the extract of S2 cells expressing *Hymenoptaecin* (Figure 5C).

## Discussion

### *T. mercedesae* Functions as a Vector for DWV Without Active Replication Inside Mite

Our results show that the artificial infestation of honey bee pupae with *T. mercedesae* increases DWV copy number in pupae, but also transfers the variant present in the mite to the pupa. These results demonstrate that *T. mercedesae* functions as a bona fide vector for DWV and also stimulates replication of the endogenous DWV in honey bee pupae. These properties are similar to those of *V. destructor* (Kuster et al., 2014). Replication of the endogenous DWV can be induced by wounds caused by mite feeding. Consistent with previous reports for both *T. mercedesae* and *V. destructor* (Wu et al., 2017; Posada-Florez et al., 2019), the copy number of DWV could exceed 10^6^. However, we found that DWV had little effect on the transcriptome of *T. mercedesae.* Thus, DWV does not appear to significantly affect the fitness of mites. In fact, most DWV is present in the midgut lumen of mite, as previously reported with *V. destructor* (Zhang et al., 2007; Santillan-Galicia et al., 2008). Furthermore, DWV RdRP was not detected in the mites with high DWV loads by western blotting. These results demonstrate that most of the DWV in the mite is derived from infested honey bee pupae and does not actively replicate inside the mite cells. Furthermore, the large difference in codon usage between DWV and *T. mercedesae* is unlikely to support the active translation of DWV proteins. Proteomic analysis of *T. mercedesae* and *V. destructor* also failed to detect the non-structural proteins of honey bee viruses (Erban et al., 2015; Dong et al., 2017). Previous studies have suggested that the presence of a negative strand of DWV genome RNA in *V. destructor* is evidence of viral replication (Ongus et al., 2004; Yue and Genersch, 2005). However, this was recently questioned (Posada-Florez et al., 2019). Therefore, our results do not support that the mites actively amplify the specific variant of DWV. The six pupa-mite pairs (Bee/Mite-3, 5, 10, 11, 13, and 15) contained four variants originally derived from the mites, indicating that they were specifically amplified in the honey bee over the endogenous variants. These results suggest that there is a mechanism to amplify the specific variant introduced by mite in honey bee. Since *T. mercedesae* is capable of transferring the associated variant to honey bee pupae, DWV must be present in the mite salivary gland. Although we did not detect DWV in the salivary gland by immunostaining of the sections, DWV has been previously detected in the salivary gland of *V. destructor* using mass spectrometry (Zhang and Han, 2019). The migration mechanism of DWV from the midgut to the salivary gland in the mites could be common in Arthropod-borne viruses, such as dengue virus and Zika virus in mosquitos (Cui et al., 2019). It is also possible that selection of DWV variants may occur during migration from the midgut to the salivary gland. The mechanism of DWV transmission by honey bee mites as well as the virus/mite relationship could also help to understand the mechanisms of tick-borne viral diseases, for example, Tick-borne meningoencephalitis (Mansfield et al., 2009), and mosquito-borne virus diseases.

### Downregulation of Honey Bee Mite Reproduction by Host Immune Effector

Consistent with previous reports for *V. destructor* (Gregorc et al., 2012; Kuster et al., 2014), we found that *Defensin-1* and *Hymenoptaecin* mRNAs were induced by *Tropilaelapse* mite infestation, but by different mechanisms. Our results demonstrate that *Defensin-1* and *Hymenoptaecin* are induced by DWV replication and mite feeding, respectively. Since Defensin-1 and Hymenoptaecin were suggested to be under the control of the Toll and Imd pathways, respectively (Schlüns and Crozier, 2007; Lourenço et al., 2018), these immune signaling pathways would be independently activated by the above events. We also demonstrated a negative correlation between *Hymenoptaecin* mRNA and *TmVg-1* or *TmVg-2* mRNA, and further demonstrated that Hymenoptaecin in S2 cell extract down-regulates expression of *TmVg-2* mRNA. Vg is a precursor for the major yolk protein vitellin, providing essential nutrients for the embryo (Tufail and Takeda, 2008). Thus, Vg and Vitellogenin receptor are essential for the reproduction of mites, such as *Panonychus citri* (Ali et al., 2017). Accordingly, in *V. destructor*, high levels of *VdVg-1* and *VdVg-2* mRNAs are present at the oviposition stage (Cabrera Cordon et al., 2013; McAfee et al., 2017). A previous study also reported a negative association between the reproductive capability of *V. destructor* and honey bee immune gene mRNAs, such as *Relish*, *PGRP-S1*, and *Hymenoptaecin* (Kuster et al., 2014). Hymenoptaecin is an AMP that was originally identified as a small positively-charged peptide capable of killing microorganisms by targeting the negatively charged membranes (Casteels et al., 1993). However, the physiological roles of AMPs were not precisely characterized, and recent studies have revealed that they are also critical for tumor elimination, brain function, neurodegeneration, and aging (Hanson and Lemaitre, 2019). Since the Imd pathway is involved in processes that include viral defense, resistance to dessication, resistance to oxygen stress, and autophagy (Zhai et al., 2018), AMPs as well as the other downstream effectors could be involved in these processes. Thus, it is possible that Hymenoptaecin affects the mite tissue(s) to decrease the amount of *Vg* mRNA. Nevertheless, the mechanism of downregulation as well as the tissues synthesizing Vg remains to be elucidated. Hymenoptaecin can modulate specific cell signaling pathways in mite cells by indirect activation of receptors by displacing ligands, altering membrane microdomains, or directly acting as an alternate ligand. Given the present observation that Hymenoptaecin expressed in S2 cells failed to repress *TmVg-1* mRNA, it could be under the control of other downstream effectors of the Imd pathway. Hymenoptaecin induced by mite feeding could exert negative feedback on mite reproduction by repressing Vg synthesis. In fact, fecundity of both *T. mercedesae* and *V. destructor* is low (<3 and <7, respectively per reproductive cycle) and significant fractions of non-reproductive mites have also been described (Nazzi and Le Conte, 2016; Chantawannakul et al., 2018). This equilibrium between the host (honey bee) and parasite (mite) could be established by the interaction between Hymenoptaecin and mite Vg. Our study should help further explorations of the novel physiological roles of AMPs in honey bees and other insects.

## Data Availability Statement

The datasets presented in this study can be found in online repositories. The names of the repository/repositories and accession number(s) can be found in the article/ Supplementary Material.

## Author Contributions

TK conceived and designed the research strategy. YW, QL, and BW performed the research. YW, MK, and TK analyzed the data and drafted and edited the manuscript.

## Conflict of Interest

The authors declare that the research was conducted in the absence of any commercial or financial relationships that could be construed as a potential conflict of interest.

## Footnotes

1 https://www.ncbi.nlm.nih.gov/genome/53919?genome_assembly_id=313451

## References

[B1] Aizen M. A. Harder L. D. (2009). The global stock of domesticated honey bees is growing slower than agricultural demand for pollination. *Curr. Biol.* 19 915–918. 10.1016/j.cub.2009.03.071 19427214

[B2] Ali M. W. Zhang Z. Y. Xia S. Zhang H. (2017). Biofunctional analysis of Vitellogenin and Vitellogenin receptor in citrus red mites, *Panonychus citri* by RNA interference. *Sci. Rep.* 7:16123. 10.1038/s41598-017-16331-3 29170435 PMC5701056

[B3] Anders S. Pyl P. T. Huber W. (2015). HTSeq–a Python framework to work with high-throughput sequencing data. *Bioinformatics* 31 166–169. 10.1093/bioinformatics/btu638 25260700 PMC4287950

[B4] Athey J. Alexaki A. Osipova E. Rostovtsev A. Santana-Quintero L. V. Katneni U. (2017). A new and updated resource for codon usage tables. *BMC Bioinformatics* 18:391. 10.1186/s12859-017-1793-7 28865429 PMC5581930

[B5] Bahar A. A. Ren D. (2013). Antimicrobial peptides. *Pharmaceuticals* 6 1543–1575. 24287494 10.3390/ph6121543 PMC3873676

[B6] Cabrera A. R. Shirk P. D. Teal P. E. A. (2017). A feeding protocol for delivery of agents to assess development in *Varroa mites*. *PLoS One* 12:e0176097. 10.1371/journal.pone.0176097 28448606 PMC5407785

[B7] Cabrera Cordon A. R. Shirk P. D. Duehl A. J. Evans J. D. Teal P. E. (2013). Variable induction of vitellogenin genes in the varroa mite, Varroa destructor (Anderson & Trueman), by the honeybee, *Apis mellifera* L, host and its environment. *Insect. Mol. Biol.* 22 88–103. 10.1111/imb.12006 23331492

[B8] Casteels P. Ampe C. Jacobs F. Tempst P. (1993). Functional and chemical characterization of Hymenoptaecin, an antibacterial polypeptide that is infection-inducible in the honeybee (*Apis mellifera*). *J. Biol. Chem.* 268 7044–7054. 8463238

[B9] Chantawannakul P. Cutler R. W. (2008). Convergent host-parasite codon usage between honeybee and bee associated viral genomes. *J. Invertebr. Pathol.* 98 206–210. 10.1016/j.jip.2008.02.016 18397791

[B10] Chantawannakul P. Ramsey S. vanEngelsdorp D. Khongphinitbunjong K. Phokasem P. (2018). Tropilaelaps mite: an emerging threat to European honey bee. *Curr. Opin. Insect. Sci.* 26 69–75. 10.1016/j.cois.2018.01.012 29764663

[B11] Cui Y. Grant D. G. Lin J. Yu X. Franz A. W. E. (2019). Zika Virus Dissemination from the midgut of *Aedes aegypti* is facilitated by bloodmeal-mediated structural modification of the midgut basal lamina. *Viruses* 11:1056 10.3390/v11111056 PMC6893695 31739432

[B12] de Miranda J. R. Genersch E. (2010). Deformed wing virus. *J. Invertebr. Pathol.* 103, S48–S61. 10.1016/j.jip.2009.06.012 19909976

[B13] Dong X. Armstrong S. D. Xia D. Makepeace B. L. Darby A. C. Kadowaki T. J. G. (2017). Draft genome of the honey bee ectoparasitic mite, Tropilaelaps mercedesae, is shaped by the parasitic life history. *Gigascience* 6:gix008. 10.1093/gigascience/gix008 28327890 PMC5467014

[B14] Edgar R. C. (2004). MUSCLE: multiple sequence alignment with high accuracy and high throughput. *Nucleic Acids Res.* 32 1792–1797. 10.1093/nar/gkh340 15034147 PMC390337

[B15] Egekwu N. I. Posada F. Sonenshine D. E. Cook S. (2018). Using an in vitro system for maintaining *Varroa destructor* mites on *Apis mellifera* pupae as hosts: studies of mite longevity and feeding behavior. *Exp. Appl. Acarol.* 74 301–315. 10.1007/s10493-018-0236-0 29511937

[B16] Erban T. Harant K. Hubalek M. Vitamvas P. Kamler M. Poltronieri P. (2015). In-depth proteomic analysis of *Varroa destructor*: detection of DWV-complex, ABPV, VdMLV and honeybee proteins in the mite. *Sci. Rep.* 5 1–16. 10.1038/srep13907 26358842 PMC4566121

[B17] Forsgren E. De Miranda J. R. Isaksson M. Wei S. Fries I. J. E. Acarology A. (2009). Deformed wing virus associated with *Tropilaelaps mercedesae* infesting European honey bees (*Apis mellifera*). *Exp. Appl. Acarol.* 47 87–97. 10.1007/s10493-008-9204-4 18941909

[B18] Goulson D. Nicholls E. Botías C. Rotheray E. L. (2015). Bee declines driven by combined stress from parasites, pesticides, and lack of flowers. *Science* 347:1255957. 10.1126/science.1255957 25721506

[B19] Gregorc A. Evans J. D. Scharf M. Ellis J. D. (2012). Gene expression in honey bee (*Apis mellifera*) larvae exposed to pesticides and *Varroa mites* (*Varroa destructor*). *J. Insect. Physiol.* 58 1042–1049. 10.1016/j.jinsphys.2012.03.015 22497859

[B20] Hanson M. A. Lemaitre B. (2019). New insights on Drosophila antimicrobial peptide function in host defense and beyond. *Curr. Opin. Immunol.* 62 22–30. 10.1016/j.coi.2019.11.008 31835066

[B21] Highfield A. C. El Nagar A. Mackinder L. C. Noël L. M. Hall M. J. Martin S. J. (2009). Deformed wing virus implicated in overwintering honeybee colony losses. *Appl. Environ. Microbiol.* 75 7212–7220. 10.1128/AEM.02227-09 19783750 PMC2786540

[B22] Jiang P. Liu Y. Ma H. C. Paul A. V. Wimmer E. (2014). Picornavirus morphogenesis. *Microbiol. Mol. Biol. Rev.* 78 418–437. 25184560 10.1128/MMBR.00012-14 PMC4187686

[B23] Khongphinitbunjong K. De Guzman L. I. Tarver M. R. Rinderer T. E. Chantawannakul P. (2015). Interactions of *Tropilaelaps mercedesae*, honey bee viruses and immune response in *Apis mellifera*. *J. Apic. Res.* 54 40–47.

[B24] Kim D. Langmead B. Salzberg S. L. (2015). HISAT: a fast spliced aligner with low memory requirements. *Nat. Methods* 12:357. 10.1038/nmeth.3317 25751142 PMC4655817

[B25] Klein A. M. Vaissière B. E. Cane J. H. Steffan-Dewenter I. Cunningham S. A. Kremen C. (2007). Importance of pollinators in changing landscapes for world crops. *Proc. Biol. Sci.* 274 303–313. 10.1098/rspb.2006.3721 17164193 PMC1702377

[B26] Koelle M. R. Talbot W. S. Segraves W. A. Bender M. T. Cherbas P. Hogness D. S. (1991). The Drosophila EcR gene encodes an ecdysone receptor, a new member of the steroid receptor superfamily. *Cell* 67 59–77. 10.1016/0092-8674(91)90572-g 1913820

[B27] Kumar S. Stecher G. Tamura K. (2016). MEGA7: molecular evolutionary genetics analysis Version 7.0 for bigger datasets. *Mol. Biol. Evol.* 33 1870–1874. 10.1093/molbev/msw054 27004904 PMC8210823

[B28] Kuster R. D. Boncristiani H. F. Rueppell O. (2014). Immunogene and viral transcript dynamics during parasitic Varroa destructor mite infection of developing honey bee (*Apis mellifera*) pupae. *J. Exp. Biol.* 217(Pt 10), 1710–1718. 10.1242/jeb.097766 24829325

[B29] Li H. Handsaker B. Wysoker A. Fennell T. Ruan J. Homer N. (2009). The Sequence Alignment/Map format and SAMtools. *Bioinformatics* 25 2078–2079. 10.1093/bioinformatics/btp352 19505943 PMC2723002

[B30] Lourenço A. P. Florecki M. M. Simões Z. L. P. Evans J. D. (2018). Silencing of *Apis mellifera* dorsal genes reveals their role in expression of the antimicrobial peptide defensin-1. *Insect. Mol. Biol.* 27 577–589. 10.1111/imb.12498 29663584

[B31] Mansfield K. L. Johnson N. Phipps L. P. Stephenson J. R. Fooks A. R. Solomon T. (2009). Tick-borne encephalitis virus - a review of an emerging zoonosis. *J. Gen. Virol.* 90(Pt 8), 1781–1794. 10.1099/vir.0.011437-0 19420159

[B32] Martin S. Highfield A. Brettell L. Villalobos E. Budge G. Powell M. (2012). Global honey bee viral landscape altered by a parasitic mite. *Science* 336 1304–1306. 10.1126/science.1220941 22679096

[B33] McAfee A. Chan Q. W. T. Evans J. Foster L. J. (2017). A Varroa destructor protein atlas reveals molecular underpinnings of developmental transitions and sexual differentiation. *Mol. Cell Proteom.* 16 2125–2137. 10.1074/mcp.RA117.000104 PMC5724176 28867676

[B34] Nazzi F. Le Conte Y. (2016). Ecology of *Varroa destructor*, the major ectoparasite of the western honey bee. *Apis mellifera*. *Annu. Rev. Entomol.* 61 417–432. 10.1146/annurev-ento-010715-023731 26667378

[B35] Ongus J. R. Peters D. Bonmatin J.-M. Bengsch E. Vlak J. M. van Oers M. M. (2004). Complete sequence of a picorna-like virus of the genus Iflavirus replicating in the mite *Varroa destructor*. *J. Gen. Virol.* 85 3747–3755. 10.1099/vir.0.80470-0 15557248

[B36] Posada-Florez F. Childers A. K. Heerman M. C. Egekwu N. I. Cook S. C. Chen Y. (2019). Deformed wing virus type A, a major honey bee pathogen, is vectored by the mite *Varroa destructor* in a non-propagative manner. *Sci. Rep.* 2:12445 10.1038/s41598-019-47447-3 PMC6712216 31455863

[B37] Ramsey S. D. Ochoa R. Bauchan G. Gulbronson C. Mowery J. D. Cohen A. (2019). feeds primarily on honey bee fat body tissue and not hemolymph. *Proc. Natl. Acad. Sci. U.S.A.* 116 1792–1801. 10.1073/pnas.1818371116 30647116 PMC6358713

[B38] Robinson M. D. McCarthy D. J. Smyth G. K. (2010). edgeR: a Bioconductor package for differential expression analysis of digital gene expression data. *Bioinformatics* 26 139–140. 10.1093/bioinformatics/btp616 19910308 PMC2796818

[B39] Rosenkranz P. Aumeier P. Ziegelmann B. (2010). Biology and control of Varroa destructor. *J. Invertebr. Pathol.* 103, S96–S119. 10.1016/j.jip.2009.07.016 19909970

[B40] Ryabov E. V. Wood G. R. Fannon J. M. Moore J. D. Bull J. C. Chandler D. (2014). A virulent strain of deformed wing virus (DWV) of honeybees (*Apis mellifera*) prevails after *Varroa destructor*-mediated, or in vitro, transmission. *PLoS Pathog.* 10:e1004230. 10.1371/journal.ppat.1004230 24968198 PMC4072795

[B41] Santillan-Galicia M. T. Carzaniga R. Ball B. V. Alderson P. G. (2008). Immunolocalization of deformed wing virus particles within the mite Varroa destructor. *J. Gen. Virol.* 89 1685–1689. 10.1099/vir.0.83223-0 18559939

[B42] Schlüns H. Crozier R. H. (2007). Relish regulates expression of antimicrobial peptide genes in the honeybee, *Apis mellifera*, shown by RNA interference. *Insect. Mol. Biol.* 16 753–759. 10.1111/j.1365-2583.2007.00768.x 18093004

[B43] Shen M. Yang X. Cox-Foster D. Cui L. (2005). The role of varroa mites in infections of Kashmir bee virus (KBV) and deformed wing virus (DWV) in honey bees. *Virology* 342 141–149. 10.1016/j.virol.2005.07.012 16109435

[B44] Tentcheva D. Gauthier L. Bagny L. Fievet J. Dainat B. Cousserans F. (2006). Comparative analysis of deformed wing virus (DWV) RNA in Apis mellifera and Varroa destructor. *Apidologie* 37, 41–50. 10.1051/apido:2005057

[B45] Tufail M. Takeda M. (2008). Molecular characteristics of insect vitellogenins. *J. Insect. Physiol.* 54 1447–1458. 18789336 10.1016/j.jinsphys.2008.08.007

[B46] Wu Y. Dong X. Kadowaki T. (2017). Characterization of the Copy Number and Variants of Deformed Wing Virus (DWV) in the pairs of honey bee pupa and infesting. *Front. Microbiol.* 8:1558. 10.3389/fmicb.2017.01558 28878743 PMC5572262

[B47] Wu Y. Liu Q. Weiss B. Kaltenpoth M. Kadowaki T. (2020). Honey bee suppresses the parasitic mite Vitellogenin by antimicrobial peptide. *bioRxiv [Preprint]* 10.1101/2020.02.29.970913v1 PMC7261897 32523577

[B48] Yue C. Genersch E. (2005). RT-PCR analysis of Deformed wing virus in honeybees (*Apis mellifera*) and mites (*Varroa destructor*). *J. Gen. Virol.* 86(Pt 12), 3419–3424. 10.1099/vir.0.81401-0 16298989

[B49] Zhai Z. Huang X. Yin Y. (2018). Beyond immunity: the Imd pathway as a coordinator of host defense, organismal physiology and behavior. *Dev. Comp. Immunol.* 83 51–59. 10.1016/j.dci.2017.11.008 29146454

[B50] Zhang Q. Ongus J. R. Boot W. J. Calis J. Bonmatin J.-M. Bengsch E. (2007). Detection and localisation of picorna-like virus particles in tissues of Varroa destructor, an ectoparasite of the honey bee. *Apis mellifera*. *J. Invertebr. Pathol.* 96 97–105. 10.1016/j.jip.2007.03.019 17574570

[B51] Zhang Y. Han R. (2019). Insight into the salivary secretome of *Varroa destructor* and salivary toxicity to *Apis cerana*. *J. Econ. Entomol.* 112 505–514. 10.1093/jee/toy224 30219905

