# Supplementary material for: Honey Bee Suppresses the Parasitic Mite Vitellogenin by Antimicrobial Peptide
Source: Front Microbiol. 2020 May 25;11:1037. doi: 10.3389/fmicb.2020.01037 (PMC7261897; doi:10.3389/fmicb.2020.01037)
Supplement: Supplementary file 1 [file Data_Sheet_1.pdf]

Figure S1 Presence of single and multiple DWV variants in honey bee pupae and the infesting *T. mercedesae*

Representative Sanger sequencing electropherograms of the RT-PCR products show (A) the presence of single variant in pupa and multiple variants in the infesting mite (Bee/Mite-13), (B) the presence of multiple variants in pupa and single variant in the infesting mite (Bee/Mite-12), and (C) the presence of multiple variants in both pupa and the infesting mite (Bee/Mite-2). Only single peaks are present at all positions for the single variant; however, two peaks (show by asterisks) are present at several positions for the multiple variant.

Figure S2 Immunostaining of *T. mercedesae* sections by the pre-immune serum

Transverse sections of *T. mercedesae* were immunostained by the pre-immune serum (A, D, G, and J) as well as DAPI (B, E, H, and K). The merged images (Merged: C, F, I, and L) are also shown. There is no specific staining in all sections. The anterior to posterior direction of mite body is also shown.

Figure S3 Phylogeny of *T. mercedesae* and *V. destructor* vitellogenins (Vgs)

Phylogenetic tree of five and three Vgs of *T. mercedesae* and *V. destructor* was constructed by maximum-likelihood method. Bootstrap values are shown at the corresponding node of each branch.

Figure S4 Expression of Hymenoptaecin in S2 cells

Lysates of S2 cells transfected with either empty vector (Mock) or *Hymenoptaecin*-expressing construct were analyzed by western blot using anti-His tag antibody. The size of protein molecular weight marker (MW) is at the left. A major 15kDa protein is present in *Hymenoptaecin*-expressing S2 cells.

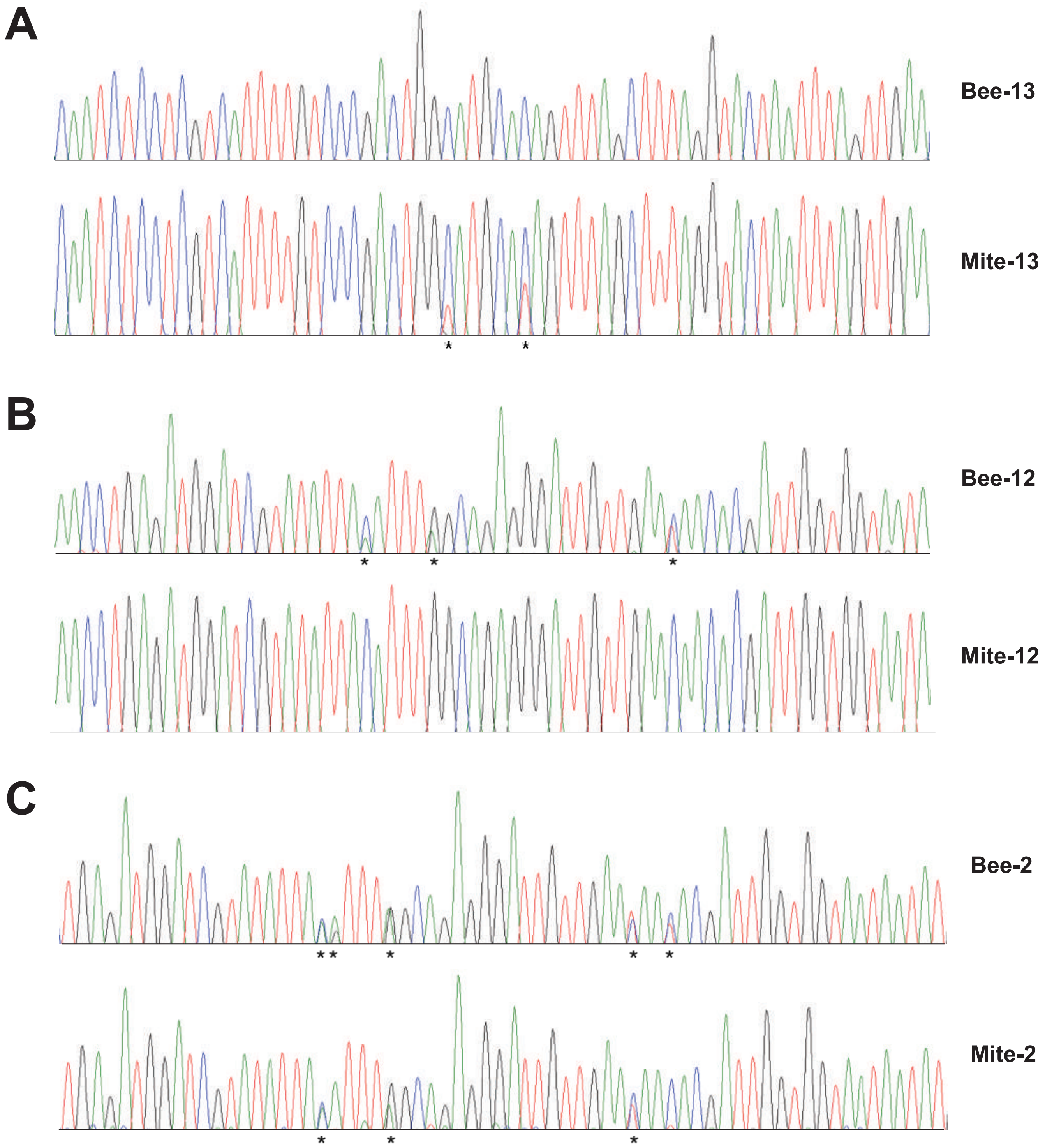
A
Bee-13
Mite-13
B
Bee-12
Mite-12
C
Bee-2
Mite-2

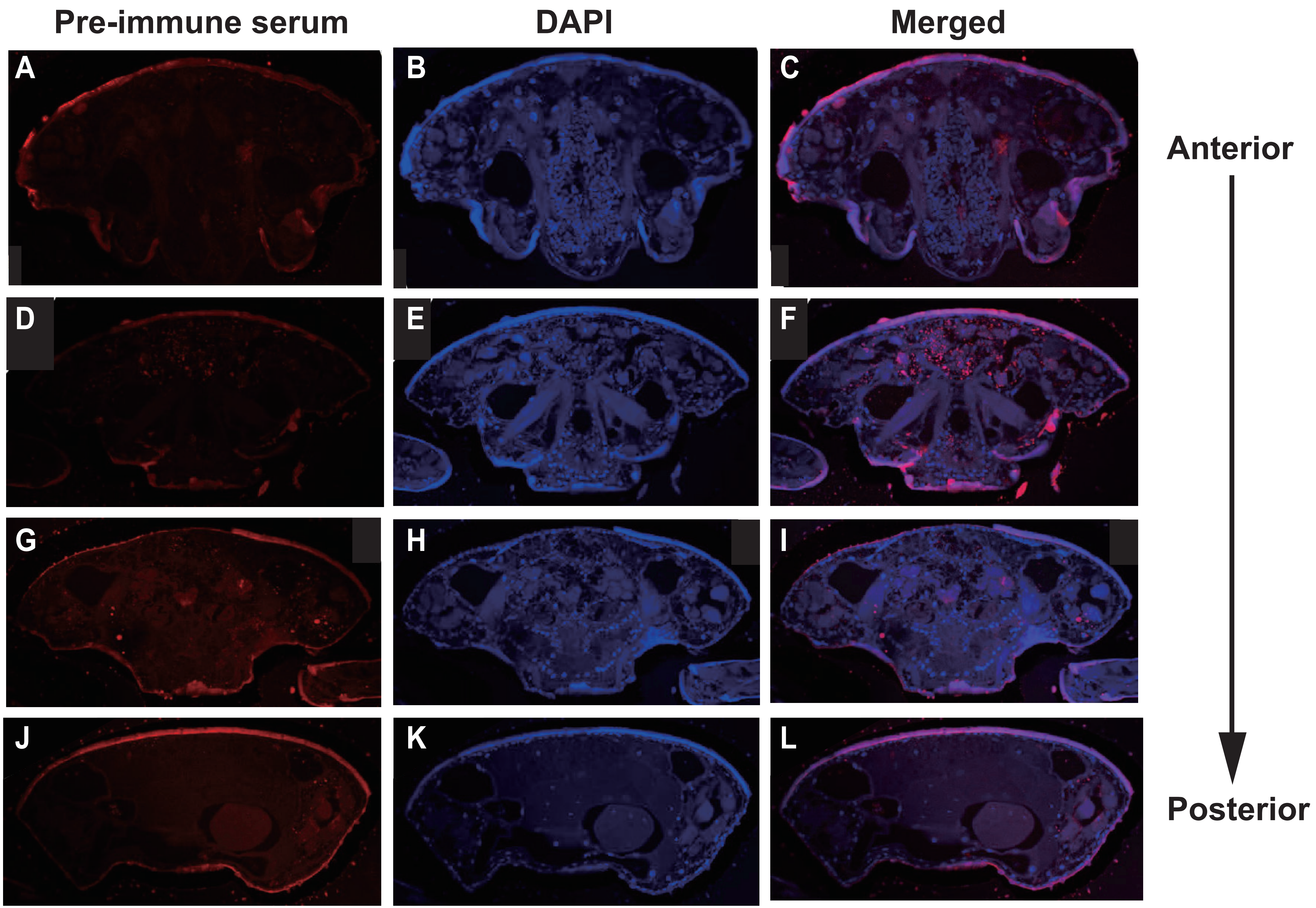

Pre-immune serum
DAPI
Merged
A
B
C
D
E
F
G
H
I
J
K
L
Anterior
Posterior

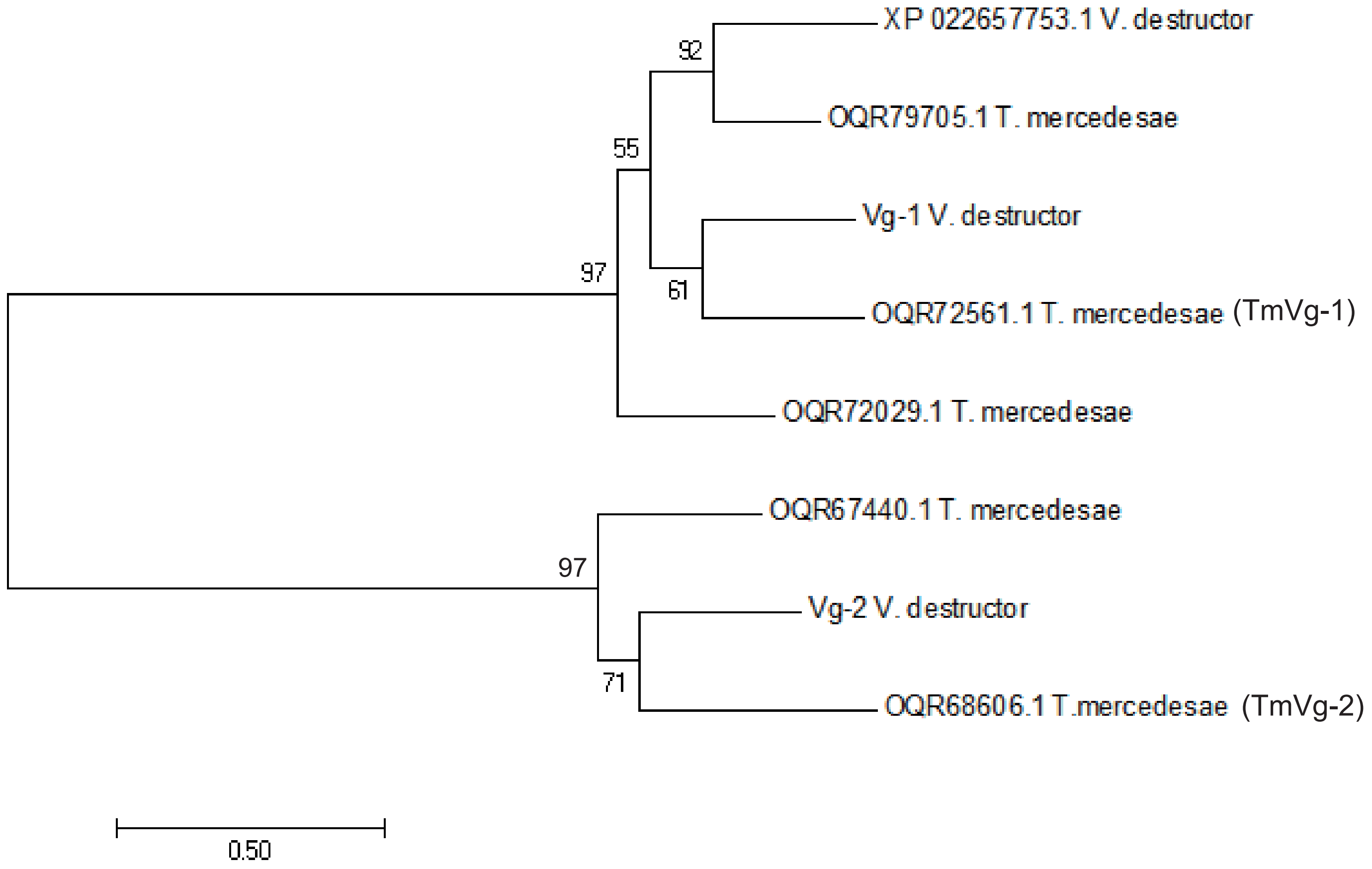
XP 022657753.1 V. destructor
OQR79705.1 T. mercedesae
Vg-1 V. destructor
OQR72561.1 T. mercedesae (TmVg-1)
OQR72029.1 T. mercedesae
OQR67440.1 T. mercedesae
Vg-2 V. destructor
OQR68606.1 T.mercedesae (TmVg-2)
92
55
61
97
97
71
0.50

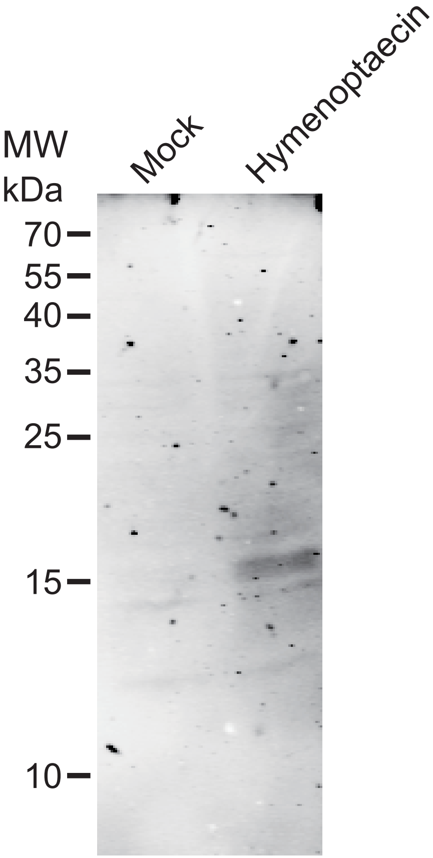
MW
kDa
Mock
Hymenoptaecin
70
55
40
35
25
15
10

Table S1

List of primers used in this study

Primers	Sequences (5' to 3')
DWV #1	(F) ATTGTGCCAGATTGGACTAC
	(R) AGATGCAATGGAGGATACAG
DWV #2	(F) TTCATTAAAGCCACCTGGAACATC
	(R) TTTCCTCATTAACTGTGTCGTTGA
A. mellifera EF-1α	(F) TGCAAGAGGCTGTTCCTGGTGA
	(R) CGAAACGCCCCAAAGGCGGA
T. mercedesae EF-1α	(F) ATTCCGGTAAGTCAACCACCAC
	(R) GCTCGGCCTTCAGTTTGTCCAA
A. mellifera 18S rRNA	(F) ACCACATCCAAGGAAGGCAG
	(R) ACTCATTCCGATTACGGGGC
T. mercedesae 18S rRNA	(F) CCTTCGGACTTACGGTGACG
	(R) TATGTGGTCGCCGTTTCTCA
Hymenoptaecin	(F) CCGACTCGTTTCCGACGAC
	(R) CGTCTCCTGTCATTCCATTC
Defensin-1	(F) GCATTTTGAGAATGAAGAACG
	(R) CAAACTGAGACAGTTAGCAG
TmVg-1	(F) GCTCTCGGTGGTCAACAGAA
	(R) TTACGTACTGGTTGGTGCCC
TmVg-2	(F) ACTGGTCAGCAGCAGTACAC
	(R) CGACGACATTTCGGCGTTAC
OQR79705.1	(F) AGCAACCGAAGCAGATGTCA
	(R) GCTCAATGGCGATCTTCACG
OQR72029.1	(F) GAGTCTTATTACAACCCTGCTGAAGAC
	(R) GAAAAGCGTCTGCATAAAAGGTCCGTG
OQR67440.1	(F) ATTAAGGCCTTCGCCAAGATCGACC
	(R) TGAGCGAGGAGCGTGAACTTCGGATC
5’-Nde I-P-domain	TTTCATATGAGGGCTAAGACAGGTTATGCACCATAT
3’-Xho I-P-domain	TTTTCTCGAGCTATTCTGGAATAGCTTCAATAAATTCAA AATC
5’-Kpn I-RdRP	AAAGGTACCACTTGTTTGCCTGTGGAAAAATGTA
3’-Hind III-RdRP	AAAAAGCTTCTAAGCAGTCTGTAACGTCCGCCACTTC

Hymenoptaecin #1	(F) TTTTTGAATTCATGAAATTCATCGTGTTGGTTCTCTTC
	(R) TTACCGGTAAATCTAAATCCACCATTTATTCCAAAGTA
Hymenoptaecin #2	(F) TTTTCTCGAGATGAAATTCATCGTGTTGGTTCTCTTC
	(R) TTTTTGATATCTCAAAATCTAAATCCACCATTTATTCCAAAGTA

Table S2

Profile of DWV infection in honey bee pupae and the infesting *Tropilaelaps* mites.

	Single variant in pupa	Multiple variants in pupa
Single variant in mite	1, 3, 4, 7, 8, 9, 10, 11, 14, 15	12
Multiple variants in mite	6, 13	2, 5

The pupa/mite pairs in the clusters with high and low copy numbers of DWV in Figure 1 are indicated by red and black letters, respectively.

Table S3

Alignment rates of RNA-seq reads to *T. mercedesae* and DWV genomes

	Alignment rate to *T. mercedesae* genome (%)	Alignment rate to DWV (%)
High-A	37.73	22.45
High-B	39.64	23.12
Low-A	80.44	0.08
Low-B	85.53	0.01

Table S4

T. mercedesae genes up-regulated by DWV

**Gene**	**Annotation**	***P*-value**	**FDR**
OQR67497.1	lipase member H-A-like [Tropilaelaps mercedesae]	1.49759E-06	0.00286
OQR72005.1	chymotrypsin elastase family member 3B-like [Tropilaelaps mercedesae]	8.45038E-06	0.01005
OQR70413.1	hypothetical protein BIW11_04159	3.43603E-05	0.03143

	[Tropilaelaps mercedesae]		
OQR68960.1	hypothetical protein BIW11_12564 [Tropilaelaps mercedesae]	4.31267E-05	0.03663
OQR77579.1	hypothetical protein BIW11_06990, partial [Tropilaelaps mercedesae]	5.29712E-05	0.042

T. mercedesae genes down-regulated by DWV

**Gene**	**Annotation**	***P*-value**	**FDR**
OQR70206.1	zinc finger protein-like [Tropilaelaps mercedesae]	1.7968E-14	2.1E-10
OQR70191.1	hypothetical protein BIW11_11788 [Tropilaelaps mercedesae]	1.85428E-08	0.00011
OQR74958.1	hypothetical protein BIW11_00866 [Tropilaelaps mercedesae]	1.68204E-06	0.00286
OQR67746.1	nose resistant to fluoxetine protein 6-like [Tropilaelaps mercedesae]	7.87212E-07	0.00286
OQR71056.1	hypothetical protein BIW11_11234 [Tropilaelaps mercedesae]	1.02906E-06	0.00286
OQR68367.1	hypothetical protein BIW11_12957 [Tropilaelaps mercedesae]	1.22238E-06	0.00286
OQR76942.1	hypothetical protein BIW11_07447 [Tropilaelaps mercedesae]	2.57456E-06	0.00383
OQR75105.1	cement protein RIM36-like [Tropilaelaps mercedesae]	6.00625E-06	0.00794
OQR79705.1	vitellogenin 1-like [Tropilaelaps mercedesae]	1.70535E-05	0.0169
OQR67513.1	Larval cuticle protein A3A-like [Tropilaelaps mercedesae]	1.69746E-05	0.0169

Table S5

Pearson correlation analysis between either *Defensin-1* or *Hymenoptaecin* mRNA and five *Vitellogenin*-like mRNAs of *T. mercedesae*

	TmVg-1	*TmVg-2*	*OQR79705.1*	*OQR72029.1*	*OQR67440.1*
Defensin-1	*r* = -0.12,	*r* = 0.08,	*r* = 0.15,	*r* = -0.1,	*r* = -0.14,

	$P < 0.67$	$P < 0.77$	$P < 0.59$	$P < 0.74$	$P < 0.61$
Hymenoptaecin	$r = -0.55$, $P < 0.04$	$r = -0.59$, $P < 0.03$	$r = -0.44$, $P < 0.1$	$r = -0.51$, $P < 0.06$	$r = -0.41$, $P < 0.14$

The correlation values and *P*-values are shown.
